# Vesical imaging reporting and data system (VI-RADS) could predict the survival of bladder-cancer patients who received radical cystectomy

**DOI:** 10.1038/s41598-023-48840-9

**Published:** 2023-12-06

**Authors:** Juntao Zhuang, Lingkai Cai, Huanyou Sun, Qikai Wu, Kai Li, Ruixi Yu, Qiang Cao, Pengchao Li, Xiao Yang, Qiang Lu

**Affiliations:** 1 Department of Urology, The First Affiliated Hospital of Nanjing Medical University, Nanjing, China; 2 Wuxi Medical Center, Nanjing Medical University, Wuxi, China

**Keywords:** Cancer, Urology

## Abstract

Vesical Imaging Reporting and Data System (VI-RADS) shows good potential in determining muscle-invasive bladder cancer (MIBC) patients. However, whether VI-RADS could predict the prognosis of radical cystectomy (RC) patients has not been reported. Our purpose is to determine whether VI-RADS contributed to predict oncologic outcomes. In this retrospective study, we analysed the information of bladder cancer patients who admitted to our centre from June 2012 to June 2022. All patients who underwent multiparametric magnetic resonance imaging (mpMRI) and underwent RC were included. VI-RADS scoring was performed by two radiologists blinded to the clinical data. Patients’ clinical features, pathology data, and imaging information were recorded. Kaplan–Meier method was used to estimate patients' overall survival (OS) and progression-free survival (PFS). Log-rank test was used to assess statistical differences. COX regression analysis was used to estimate risk factors. Ultimately, we included 219 patients, with 188 males and 31 females. The median age was 66 (IQR = 61–74.5) years. The VI-RADS scores were as follows: VI-RADS 1, 4 (1.8%); VI-RADS 2, 68 (31.1%); VI-RADS 3, 40 (18.3%); VI-RADS 4, 69 (31.5%); and VI-RADS 5, 38 (17.4%). Patients with VI-RADS ≥ 3 had poorer OS and PFS than those with VI-RADS < 3. The AUC of VI-RADS predicting 3-year OS was 0.804, with sensitivity of 0.824 and negative predictive value of 0.942. Multivariate COX analysis showed that VI-RADS ≥ 3 was risk factors for OS (HR = 3.517, *P* = 0.003) and PFS (HR = 4.175, *P* < 0.001). In the MIBC subgroup, patients with VI-RADS ≥ 4 had poorer OS and PFS. In the non-muscle invasive bladder cancer (NMIBC) subgroup, the prognosis of patients with VI-RADS ≥ 3 remained poorer. VI-RADS scores could effectively predict the survival of patients after RC.

## Introduction

Bladder cancer is the tenth most common cancer around the world, with more than 500,000 new cases and over 200,000 deaths annually^1,2^. The management of bladder-cancer patients is based on tumour invasion and histological grade. Non-muscle invasive bladder cancer (NMIBC) is primarily treated with transurethral resection of bladder tumour (TURBT), whereas muscle invasive bladder cancer (MIBC) is treated by radical cystectomy (RC)^3,4^.

Multiparametric magnetic resonance imaging (mpMRI) is a non-invasive and convenient examination method for bladder-cancer patients. It comprises of the sequence of T2-weighted images (T2W), diffusion-weighted images (DWIs), and dynamic contrast enhanced (DCE)^5^. Vesical Imaging Reporting and Data System (VI-RADS) based on the three sequences is used as an important guideline to determine clinical staging^5–7^. A meta-analysis has shown that VI-RADS score is good at detecting the muscle invasiveness of bladder cancer with AUC > 0.90 when using VI-RADS 3 or VI-RADS 4 as the cutoff value^8^.

Clinical treatment should be based on patient prognosis, which is actually the most important thing that patients consider. Several studies have shown that the five-year cancer specific survival rate of MIBC patients is about 65%^9,10^. The use of imaging examination to predict the treatment outcomes of tumour patient is emerging. Staal FCR, et al. analysed the prediction outcomes based on CT, MRI, and PET-CT and found their potential use for survival prediction^11^. Wu S et al. constructed a nomogram based on MRI to predict preoperative lymph-node metastases and provided a basis for improving outcomes^12^. Recently, in the field of bladder cancer, the use of imaging features to predict survival prognosis is also gradually emerging^13–16^. However, for VI-RADS to be extensively used in clinical practice, it must be further explored. Accordingly, the present study aimed to probe the value of VI-RADS in predicting the prognosis of bladder-cancer patients.

## Materials and methods

### Study design

We retrospectively analysed the information of bladder-cancer patients admitted to our centre from June 2012 to June 2022. All patients who underwent mpMRI and ultimately underwent RC were included. The exclusion criteria were as follows: (1) the pathology was not urothelial carcinoma, (2) the patient received neoadjuvant therapy, (3) VI-RADS could not be evaluated, and (4) the follow-up data were incomplete. The patient's age, gender, tumour size, tumour location, number of tumour lesions, pathological stage, pathological grade, lymph-node metastasis, lymphovascular invasion, postoperative adjuvant therapy, and other information were recorded. All methods were performed in accordance with the relevant guidelines and regulations.

### MpMRI and VI-RADS implementation

All patients received mpMRI on a 3.0 Tesla MRI system (SIEMENS MAGNETOM Verio or United-Imaging u770) under the preparation for an adequate bladder distention. T2W was obtained using a turbo spin-echo sequence. DWIs were obtained with b values of 0–1400 s/mm^2^. DCE images were obtained from a 3D-vibe sequence with a temporal resolution of 8.3 s. All images were reviewed by two experienced urogenital radiologists, blinded to clinical information. Different opinions between the two readers were resolved by a consensus. MpMRI images of patients prior to 2018 were excluded if the VI-RADS score could not be interpreted.

### Radical cystectomy operation

Radical cystectomies were mainly performed by Q. Lu and P.C. Li, assisted by Q. Cao and X. Yang. All procedures were followed in accordance with the guidelines.

### Pathological diagnosis

The surgical specimens were diagnosed by an attending physician in the department of pathology, and the diagnosis results were reviewed by a chief physician.

### Statistical analyses

We performed Kaplan–Meier (K–M) method to estimate patients' overall survival (OS) and progression-free survival (PFS), as well as log-rank test to assess statistical differences. To evaluate the performance of VI-RADS in predicting prognosis, a time-dependent ROC curve was constructed. The optimal cutoff value was determined based on the Youden index, and the AUC for predicting survival at 1, 3, and 5 years was calculated. Univariate COX regression analysis was conducted to estimate the hazard ratio (HR) and 95% confidence of each variable. A nomogram was plotted for clinicians to compute the survival, using “rms” package. All statistical analyses were performed in SPSS (version 26.0) and R (version 3.6.3) software, and *P* < 0.05 was considered statistically significant.

### Ethical approval and consent to participate

This study was approved by the Ethics Committees of the First Affiliated Hospital of Nanjing Medical University (2020-SR-252), and have been performed in accordance with the Declaration of Helsinki. All written informed consent to participate in the study was obtained from the patients.

## Results

**Figure 1:**
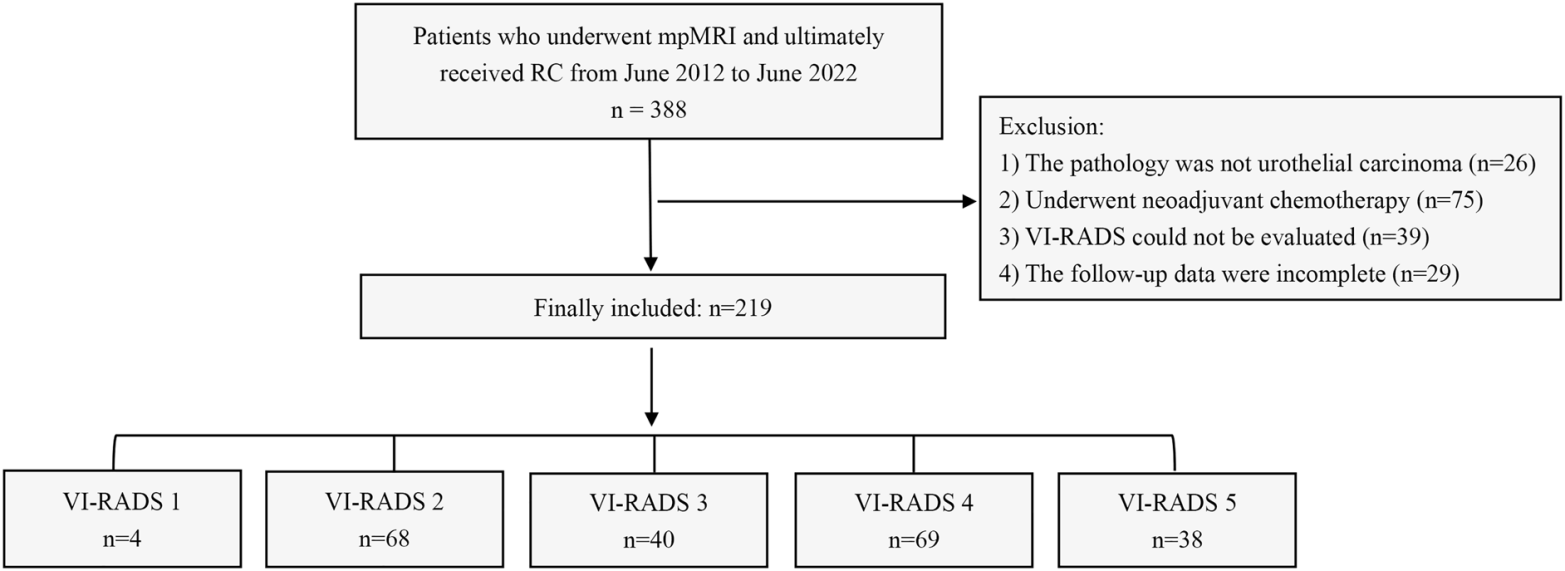
Flowchart of this study. mpMRI—multiparametric MRI; RC—radical cystectomy; VI-RADS—Vesical Imaging Reporting and Data System.

A total of 388 patients received mpMRI before RC. Based on the exclusion criteria, 219 patients were finally included (Fig. 1), including 188 (85.8%) men and 31 (14.2%) women. The median age was 66 (IQR = 61–74.5) years. VI-RADS scores well agreed between the two readers (Table S1, *Kappa* = 0.896, p < 0.001). The scores were as follows: VI-RADS 1, 4 (1.8%); VI-RADS 2, 68 (31.1%); VI-RADS 3, 40 (18.3%); VI-RADS 4, 69 (31.5%); and VI-RADS 5, 38 (17.4%). Among the patients, 126 (57.5%) patients were MIBC, and 32 (14.6%) patients had positive lymph node. The clinical and tumour characteristics of all patients are summarised in Table 1.

**Table 1:** Patient characteristics and VI-RADS evaluation.

Number	Total	VI-RADS 1	VI-RADS 2	VI-RADS 3	VI-RADS 4	VI-RADS 5
	219	4	68	40	69	38
Age						
Median Interquartile range	66 61.0–74.5	66 64.25–71.5	64 56.0–69.0	66.5 61.75–73.25	66 62.0–75.0	71.5 63.0–79.5
Gender						
Male Female	188 (85.8) 31 (14.2)	4 (100) 0	55 (80.9) 13 (19.1)	37 (92.5) 3 (7.5)	58 (84.1) 11 (15.9)	34 (89.5) 4 (10.5)
Lesion diameter						
< 3 cm ≥ 3 cm	115 (52.5) 104 (47.5)	4 (100) 0	48 (70.6) 20 (29.4)	23 (57.5) 17 (42.5)	32 (46.4) 37 (53.6)	8 (21.1) 30 (78.9)
Tumour location						
Single-walled Multi-walled	155 (70.8) 64 (29.2)	2 (50.0) 2 (50.0)	45 (66.2) 23 (33.8)	26 (65.0) 14 (35.0)	46 (66.7) 23 (33.3)	36 (94.7) 2 (5.3)
No. of lesions						
Single Multiple	122 (55.7) 97 (44.3)	2 (50.0) 2 (50.0)	34 (50.0) 34 (50.0)	20 (50.0) 20 (50.0)	37 (53.6) 32 (46.4)	29 (76.3) 9 (23.7)
Pathologic grade						
Low High	23 (10.5) 196 (89.5)	0 4 (100)	19 (27.9) 49 (72.1)	1 (2.5) 39 (97.5)	3 (4.3) 66 (95.7)	0 38 (100)
Pathologic T stage						
Ta/Tis T1 T2 T3 T4	12 (5.5) 81 (37.0) 57 (26.0) 46 (21.0) 23 (10.5)	1 (25.0) 3 (75.0) 0 0 0	8 (11.8) 49 (72.1) 7 (10.3) 1 (1.5) 3 (4.4)	1 (2.5) 17 (42.5) 16 (40.0) 3 (7.5) 3 (7.5)	2 (2.9) 10 (14.5) 33 (47.8) 16 (23.2) 8 (11.6)	0 2 (5.3) 1 (2.6) 26 (68.4) 9 (23.7)
Pathologic N stage^a^						
pN0 pN +	172 (84.3) 32 (15.7)	3 (100) 0	64 (98.5) 1 (1.5)	35 (92.1) 3 (7.9)	54 (83.1) 11 (16.9)	16 (48.5) 17 (51.5)
Lymphovascular invasion (LVI)	42 (19.2)	0	2 (2.9)	5 (12.5)	19 (27.5)	16 (42.1)
Adjuvant therapy	44 (20.1)	0	4 (5.9)	8 (20.0)	20 (29.0)	12 (31.6)

^a^15 patients were not received lymph nodes dissection.

**Figure 2:**
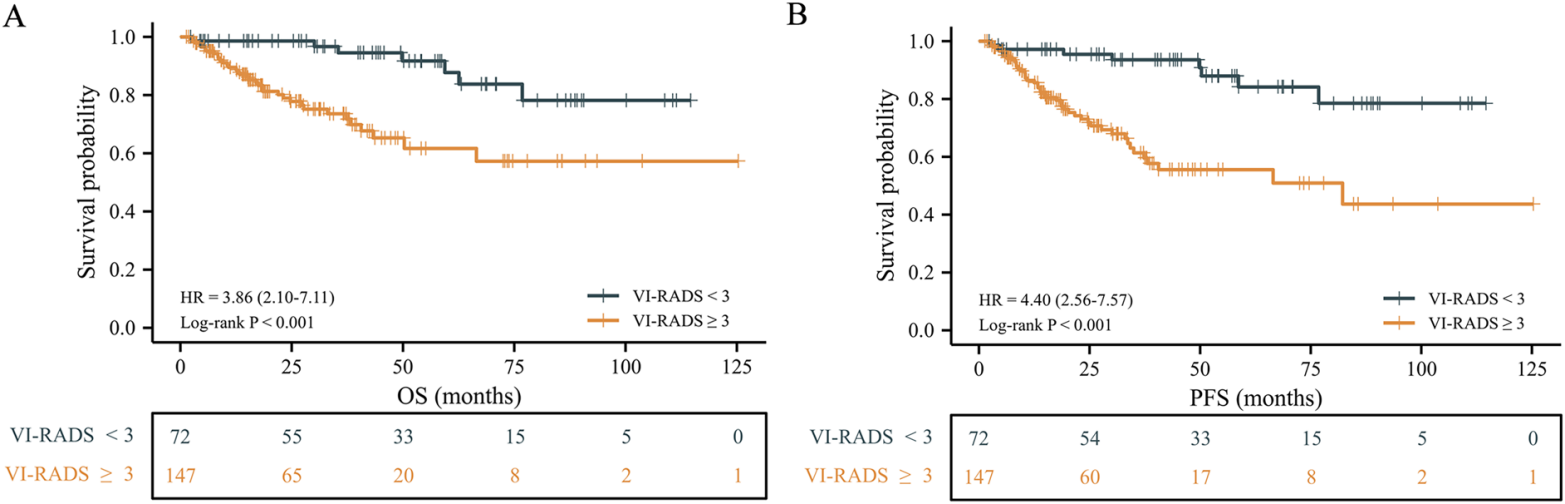
Kaplan–Meier estimates of survival stratified by VI-RADS among the 219 patients. (**A**) Overall survival. (**B**) Progression-free survival. HR, hazard ratio.

**Figure 3:**
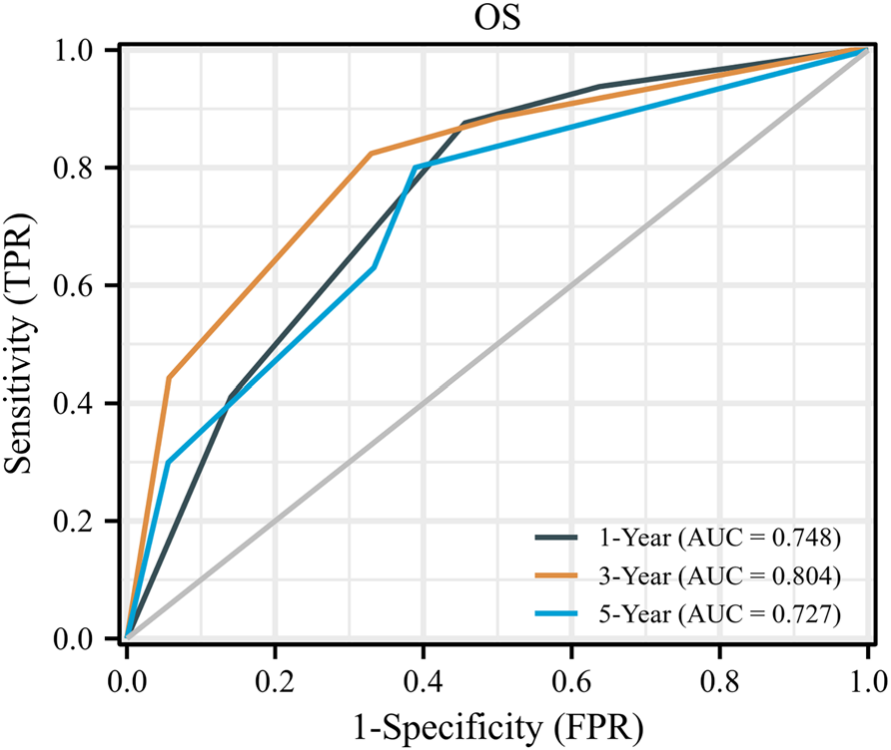
Receiver operating characteristic (ROC) curves of VI-RADS for predicting OS at 1, 3 and 5 years. AUC, area under curve.

The median follow-up was 36.5 (IQR: 30.3–43.2) months. Overall, 25 (11.4%) patients developed tumour progression, and 42 (19.2%) patients died. Patients with VI-RADS ≥ 3 had significantly lower OS and PFS than those with VI-RADS < 3 (Fig. 2A,B), with the 5-year OS of 61.7% vs. 87.7% and 5-year PFS of 55.6% vs. 84.1%. We further divided the groups into two with a cutoff of VI-RADS 4 and found the same trends (Figure S1A,B). Subsequently, we plotted time-dependent ROC curves (Fig. 3 and S2), and determined the best cutoff value according to Youden index. The AUC of VI-RADS for predicting 3-year OS was 0.804, the sensitivity was 0.824, and the negative predictive value was 0.942 (Table S2).

COX regression was used to assess the risk factors for prognosis. Univariate analysis revealed that age and VI-RADS ≥ 3 were associated with adverse OS and PFS. Tumour with multi-wall growth was a protective factor. Multivariate analysis revealed that VI-RADS ≥ 3 was risk factors for OS (HR = 3.517, *p* = 0.003) and PFS (HR = 4.175, *p* < 0.001). A nomogram based on multivariate analysis results was constructed for predicting OS (Figure S3). The COX regression results are shown in Table 2.

**Table 2:** Univariable and multivariate Cox regression models predicting OS and PFS.

Variable	OS				PFS			
	Univariate analysis		Multivariate Analysis		Univariate analysis		Multivariate analysis	
	HR (95%CI)	*P* value	HR (95%CI)	*P* value	HR (95%CI)	*P* value	HR (95%CI)	*P* value
Age	1.034 (1.004–1.064)	0.025	1.027 (0.996–1.059)	0.091	1.028 (1.002–1.055)	0.032	1.018 (0.991–1.046)	0.195
Gender (Male versus Female)	1.315 (0.517–3.348)	0.566			1.747 (0.695–4.392)	0.235		
Tumour size (≥ 3 cm versus < 3 cm)	1.593 (0.867–2.925)	0.133			1.647 (0.959–2.830)	0.071		
Tumour location (Multi versus single)	0.345 (0.145–0.820)	0.016	0.353 (0.148–0.841)	0.019	0.480 (0.241–0.958)	0.037	0.507 (0.254–1.013)	0.054
Tumour lesions (Muti versus single)	0.628 (0.334–1.182)	0.149			0.681 (0.390–1.189)	0.177		
VI-RADS (≥ 3 versus < 3)	4.078 (1.790–9.291)	0.001	3.517 (1.526–8.107)	0.003	4.680 (2.181–10.042)	< 0.001	4.175 (1.924–9.059)	< 0.001

**Figure 4:**
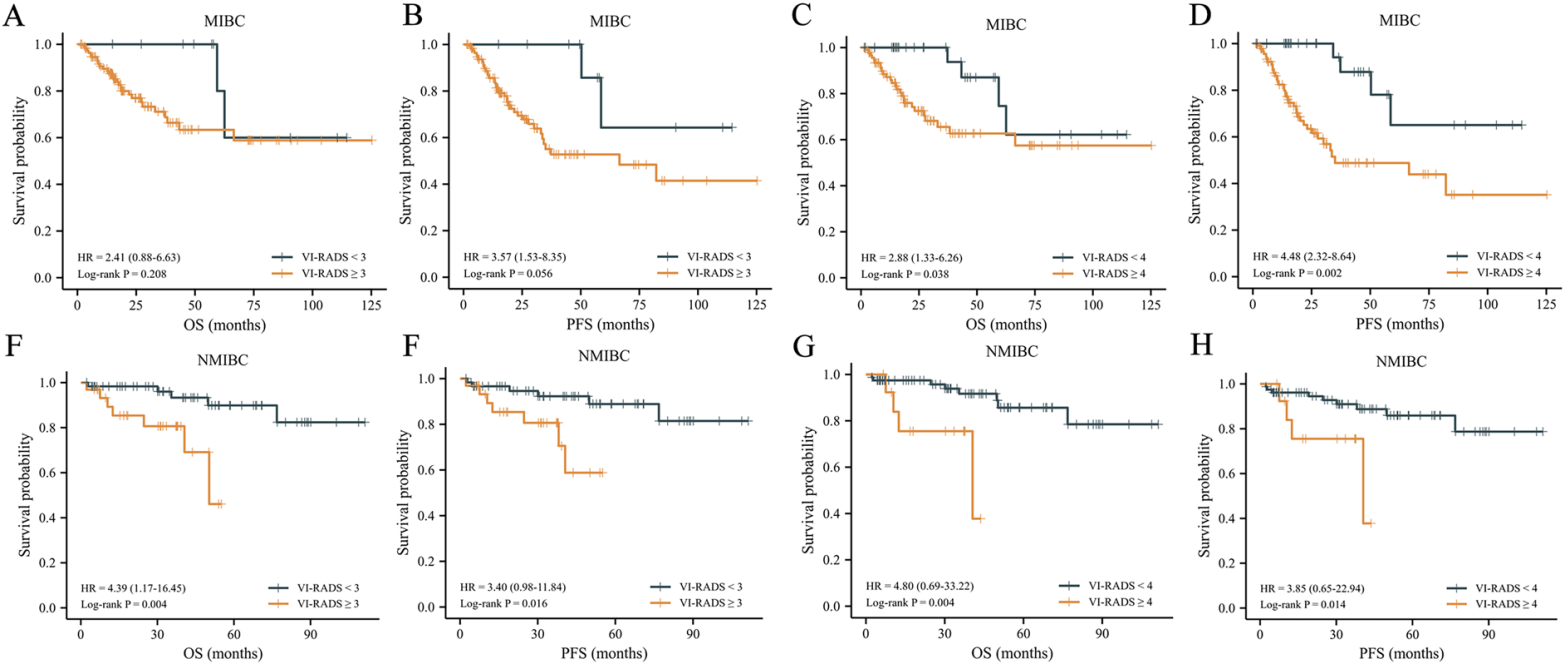
Kaplan Meier estimates of survival stratified by VI-RADS, with the cut-off of 3 or 4. (**A**) OS in MIBC subgroup, cut-off of VI-RADS 3. (**B**) PFS in MIBC subgroup, cut-off of VI-RADS 3. (**C**) OS in MIBC subgroup, cut-off of VI-RADS 4. (**D**) PFS in MIBC subgroup, cut-off of VI-RADS 4. (**E**) OS in NMIBC subgroup, cut-off of VI-RADS 3. (**F**) PFS in NMIBC subgroup, cut-off of VI-RADS 3. (**G**) OS in NMIBC subgroup, cut-off of VI-RADS 4. (**H**) PFS in NMIBC subgroup, cut-off of VI-RADS 4. MIBC—muscle invasive bladder cancer; NMIBC—non–muscle invasive bladder cancer; PFS—progression-free survival; OS—overall survival; HR—hazard ratio.

To further explore the value of VI-RADS in RC patients, we conducted subgroup analysis of each variable. In MIBC patients, no significant difference in prognosis existed between the two groups based on VI-RADS = 3 (Fig. 4A,B). Patients with VI-RADS ≥ 4 had poorer survival than those with VI-RADS < 4 (Fig. 4C,D) (5-year OS: 62.7% vs. 74.6%, 5-year PFS: 48.8% vs. 65.1%). In NMIBC patients, patients with VI-RADS ≥ 3 had significantly lower OS (5-year OS: 46.1% vs. 89.9%) and PFS (5-year PFS: 58.8% vs. 88.9%) than those with VI-RADS < 3 (Fig. 4E–4H). However, in patients with multiwall tumours, VI-RADS had little value (Figure S4A). In single- and multi-lesion patients, regardless of whether VI-RADS 3 or 4 was the cutoff, patients with VI-RADS ≥ cutoff value had poor prognosis (Figure S4B). In patients with primary lesions < 3 cm, VI-RADS 4 as the cutoff value was more valuable (Figure S4C). Moreover, we found no significant differences in OS and PFS between MIBC and NMIBC for patients with VI-RADS = 3 (Figure S5).

## Discussion

VI-RADS score system has given great reliability and clinical usefulness for distinguishing muscular invasion in bladder cancer. The AUC of VI-RADS for determining MIBC was 0.94, with a sensitivity of 0.871, specificity of 0.965, and accuracy of 0.941^17^. However, previous studies have mostly focused on the use of VI-RADS to estimate the degree of invasion. Herein, we used this system to predict the prognosis of RC patients and constructed a nomogram to broaden its clinical use.

We enrolled 219 bladder-cancer patients. Our results demonstrated a significant difference in oncology outcomes between the two groups with VI-RADS 3 or 4 as a cutoff. Specifically, with a cutoff score of 3, patients with VI-RADS < 3 had a longer mean OS time (101.5 vs. 83.9 months) and mean PFS time (100.3 vs. 72.9 months) compared with those having VI-RADS ≥ 3. This finding can serve as a theoretical basis for previous research showing that patients with VI-RADS ≥ 3 are more inclined to be MIBCs^17–19^. Conversely, Metwally MI et al. pointed that VI-RADS 4 and 5 have a high performance in defining surgical treatment, whereas VI-RADS 2 and 3 require further modification^7^. Some studies showed that in patients with intradiverticular bladder tumours, MRI features could significantly predict the overall survival^15^. In addition, Woo S, et al. enrolled 41 patients and found that VIRADS were associated with prognosis^16^.

The AUCs of VI-RADS scores in predicting the survival of RC patients all exceeded 0.7, showing its good ability. In particular, in predicting 3-year PFS, the AUC was 0.822, with a sensitivity of 0.832, specificity of 0.716, positive predictive value (PPV) of 0.512, and negative predictive value (NPV) of 0.923. With regard to the low PPV, we considered that the VI-RADS score system was designed to evaluate the primary lesion and did not take into account the status of lymph nodes. Several studies have proven that pelvic lymph-node metastasis induces cancer recurrence ^9,20^. Therefore, patients who are predicted not to recur by judging the primary lesion may have some recurrence due to the presence of positive lymph node. How to effectively predict pelvic metastatic lymph nodes before surgery is also crucial.

In the MIBC subgroup, VI-RADS with the cutoff of 4 was more valuable. A total of 22 (17.5%) MIBC patients had a VI-RADS score of 3. Accordingly, we speculated that patients with a score of 3 would have a better prognosis even if the pathology was MIBC. The K-M curve showed no significant difference in prognosis between MIBC and NMIBC when the VI-RADS score was 3. However, in the NMIBC subgroup, the prognosis of patients with VI-RADS ≥ 3 was significantly poorer than that of patients with VI-RADS < 3. Some patients with NMIBC undergo RC because of BCG-unresponsive. Ferro M, et al. showed that in patients treated with TURBT, the recurrence rate of patients with BCG-unresponsive is significantly higher than that of patients with BCG-responsive^21^. Therefore, new risk stratification based on VI-RADS may better serve NMIBC patients.

Our study also had some limitations. First, this study was a retrospective one, with existing inherent bias. Second, some of the patients (7 patients, 3.2%) in our study had received TURBT in other centres, which may increase the difficulty for judging VI-RADS scores. Even so, our study obtained some useful results and provided guidance for clinical practice in the real world.

## Conclusion

VI-RADS scores could effectively predict the survival of patients after RC. It is valuable for patients with both MIBC and NMIBC. Additionally, for patients with a VI-RADS score of 3, whether they had MIBC or NMIBC may not need to be determined.

### Supplementary Information

Supplementary Information 1.

Supplementary Information 2.

Supplementary Information 3.

## Data Availability

All data generated or analysed during this study are included in this published article and its supplementary information files. Further enquiries can be directed to the corresponding authors.
