# Supplementary material for: Vesical imaging reporting and data system (VI-RADS) could predict the survival of bladder-cancer patients who received radical cystectomy
Source: Sci Rep. 2023 Dec 6;13:21502. doi: 10.1038/s41598-023-48840-9 (PMC10700510; doi:10.1038/s41598-023-48840-9)
Supplement: Supplementary file 1 — Supplementary Information 1. [file 41598_2023_48840_MOESM1_ESM.docx]

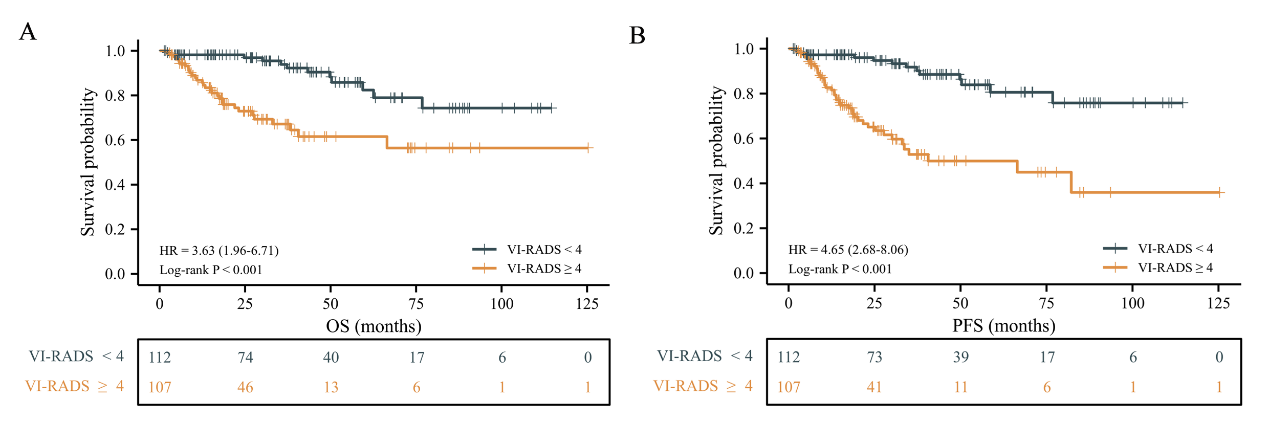


Figure S1: Kaplan Meier estimates of survival stratified by VI-RADS (with 4 as the cut-off) among the 219 patients. (A) Overall survival. (B) Progression-free survival.


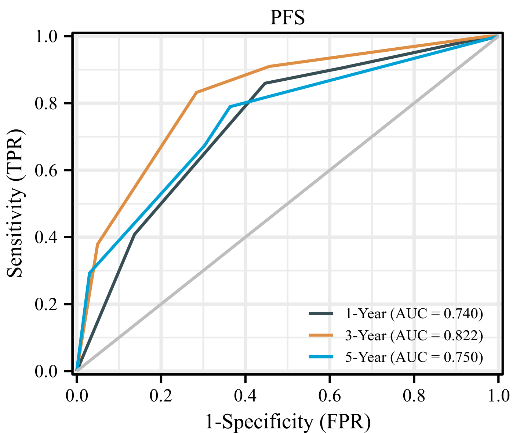


Figure S2: Receiver operating characteristic (ROC) curves of VI-RADS for predicting 1-year PFS, 3-year PFS and 5-year PFS.


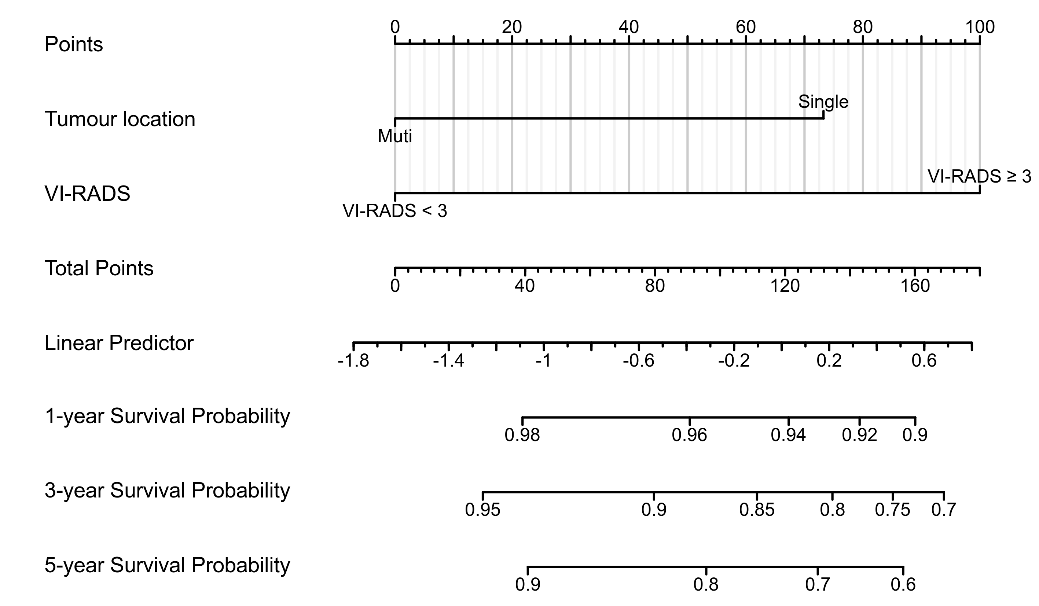


Figure S3: A nomogram based on multivariate analysis results for predicting OS.


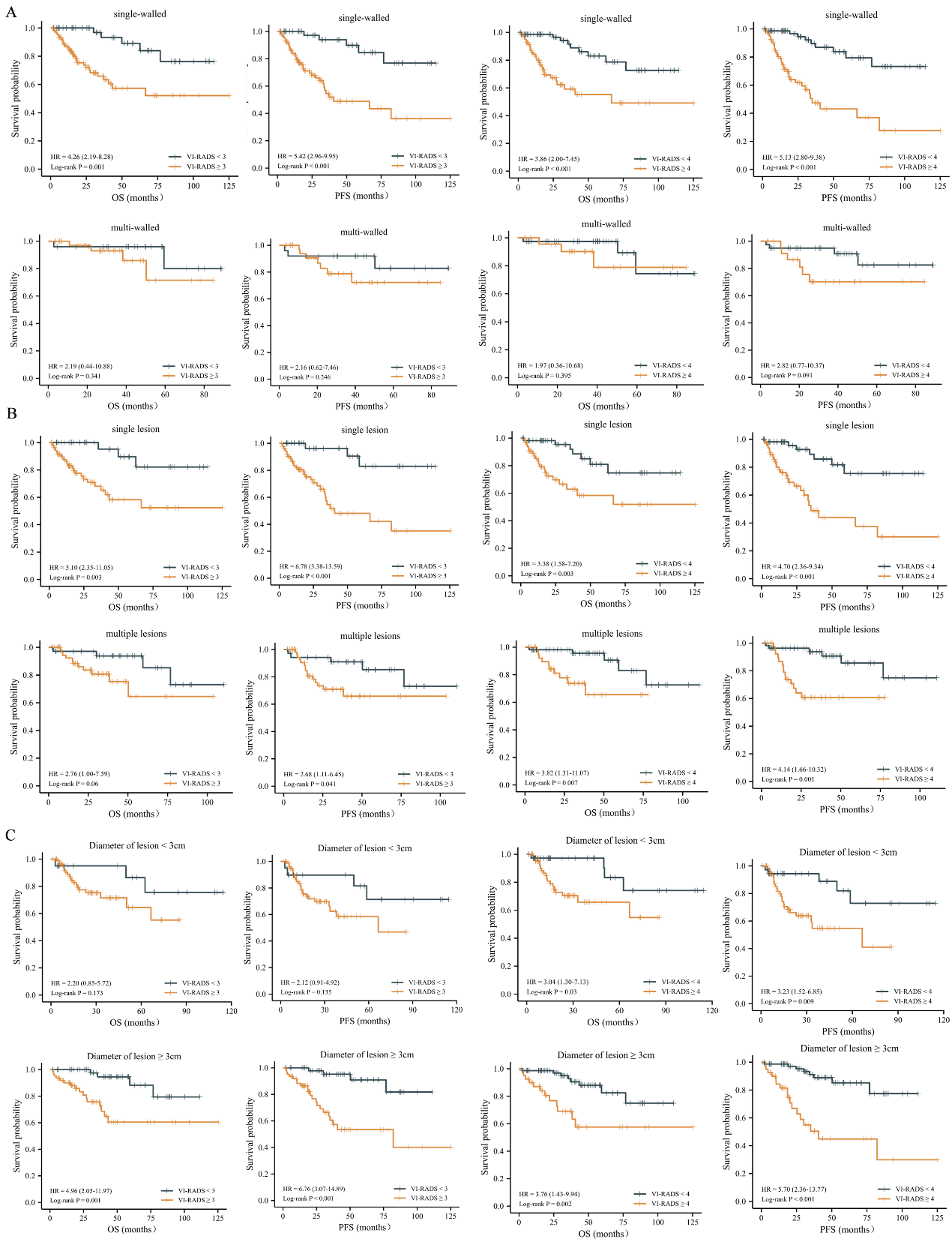


Figure S4: Kaplan Meier estimates of survival stratified by VI-RADS (with 3 or 4 as the cut-off) in different subgroups. (A) in single-walled and multi-walled. (B) in single lesion and multiple lesions. (C) in diameter of lesion ≥3cm or <3cm.


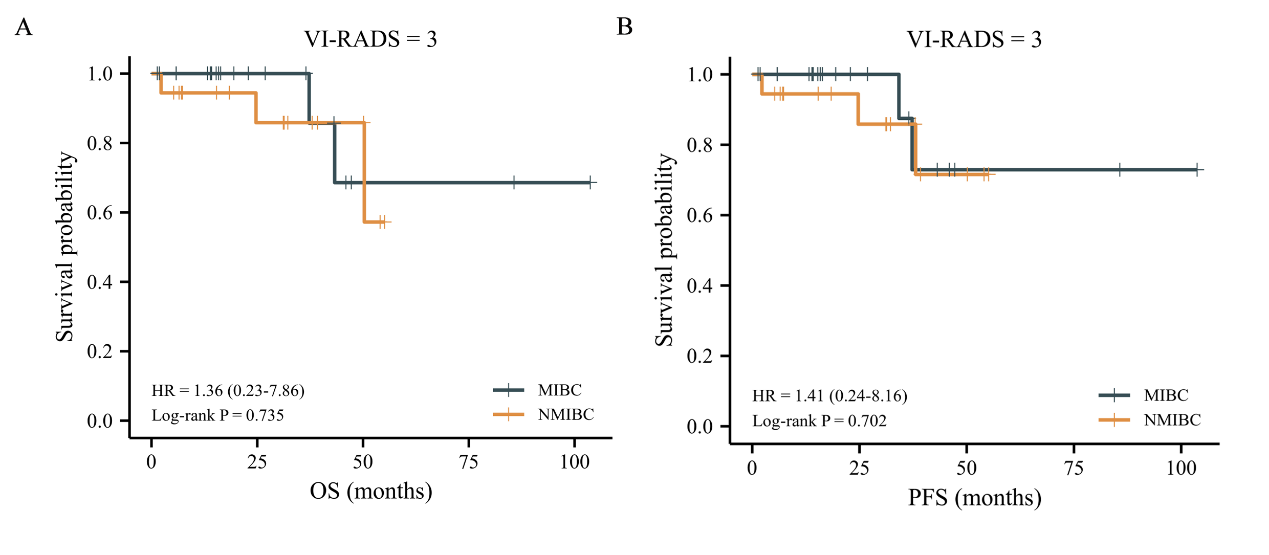


Figure S5: Kaplan Meier estimates of survival stratified by MIBC and NMIBC in patients with VI-RADS 3. (A) Overall survival. (B) Progression-free survival. HR, hazard ratio.
