# Supplementary material for: Vesical imaging reporting and data system (VI-RADS) could predict the survival of bladder-cancer patients who received radical cystectomy
Source: Sci Rep. 2023 Dec 6;13:21502. doi: 10.1038/s41598-023-48840-9 (PMC10700510; doi:10.1038/s41598-023-48840-9)
Supplement: Supplementary file 3 — Supplementary Information 3. [file 41598_2023_48840_MOESM3_ESM.docx]

Table S2 The sensitivity, specificity, positive predictive value and negative predictive value of VI-RADS for predicting OS and PFS.

|  | sensitivity | specificity | positive predictive value | negative predictive value |
| --- | --- | --- | --- | --- |
| 1-year OS | 0.876 | 0.544 | 0.134 | 0.982 |
| 3-year OS | 0.824 | 0.670 | 0.369 | 0.942 |
| 5-year OS | 0.800 | 0.611 | 0.445 | 0.887 |

|  | sensitivity | specificity | positive predictive value | negative predictive value |
| --- | --- | --- | --- | --- |
| 1-year PFS | 0.860 | 0.554 | 0.175 | 0.973 |
| 3-year PFS | 0.832 | 0.716 | 0.512 | 0.923 |
| 5-year PFS | 0.789 | 0.636 | 0.529 | 0.854 |
